# A Rare Presentation of Late-Onset Mania Following Right-Sided Lacunar Infarct

**DOI:** 10.7759/cureus.33899

**Published:** 2023-01-17

**Authors:** Apoorva Yadav, Vaishali Sehgal, Pradeep Patil, Apurva Bezalwar

**Affiliations:** 1 Psychiatry, Jawaharlal Nehru Medical College, Datta Meghe Institute of Higher Education and Research, Wardha, IND

**Keywords:** manic switch, organic mania, stroke, late onset mania, lacunar infarct

## Abstract

Stroke can result in various psychiatric disorders. It is uncommon for people to experience their first episode of mania later in life, amounting to only 1% as compared to post-stroke depression, where the incidence is comparatively high. Significant attention has been paid to the study of post-stroke depression. However, reports of manic episodes following a stroke are uncommon. Just five reported cases of mania or bipolar disorder following a cerebral infarction due to damage to the left hemisphere had been published as of late 1996. There is insufficient evidence to conclude whether late-onset mania has an organic or non-organic basis. There hasn't been a lot of research done in this area. In this report, we present a case of an elderly woman who presented with mania after being treated with an anti-depressant following a chronic cerebral infarction.

## Introduction

Mania is generally characterised by mood and behavioural abnormalities like heightened mood, grandiose beliefs, hyperactivity, and social disinhibition with a lack of insight. Late-onset mania was explained by Krauthammer and Klerman as mania caused by a neurological, metabolic, toxic illness [1]. Stroke can result in various psychiatric disorders. While depression is seen in 30-40% of stroke survivors, mania is seen in less than 1%. Mania following a stroke has been linked to activity in the frontal and basotemporal cortex [2]. It is uncommon for people to experience their first episode of mania later in life [3]. Mania is diagnosed in less than 10% of patients who suffer from depression following a brain injury, making it one of the rarest mental disorders overall. Furthermore, manic episodes are linked to more severe ischemia changes than depressive episodes. Despite the fact that the causes of mania following brain injury tend to be more severe than those of depression, the former is much less common than the latter [4]. Significant attention has been paid to the study of depression in patients secondary to cerebral insults. However, reports of manic episodes following a stroke are redundant. Following a study of 661 patients with cerebrovascular accidents, it was concluded that there were only three occurrences of post-stroke mania. Only five cases of manic or bipolar symptoms after left (dominant) hemisphere injury due to stroke had been published as of late 1996 [5]. In this report, we present a case of an elderly woman who presented with mania after being treated with an anti-depressant following a stroke.

## Case presentation

**Figure 1:**
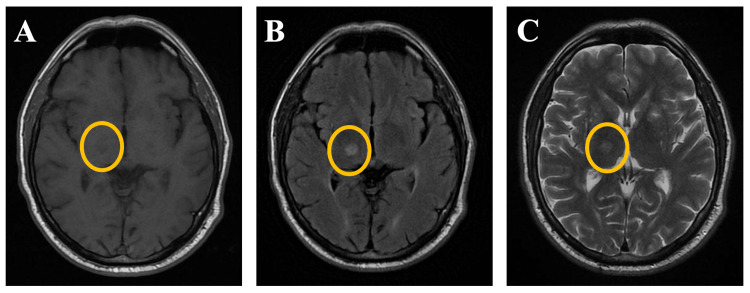
Non-contrast MRI brain images A) Axial T1 section with chronic right-sided lacunar infarct, B) FLAIR (Fluid Attenuated Inversion Recovery) showing right-sided lacunar infarct, and C) Axial T2 showing chronic right-sided lacunar infarct

A female patient who was 72 years of age came to our centre with a two months history of excessive talking, persistent irritability, abusive language, disinhibited behaviour, and a diminished need for sleep, which was indicative of a manic episode. Three months before, she had received inpatient care at another hospital after suffering repeated bouts of vomiting and generalized weakness following cataract surgery on her left eye. A previously done MRI brain report revealed a chronic infarct in the right lentiform nucleus (Figure 1).

Following her hospital discharge, she was treated by a psychiatrist with escitalopram 5 mg and clonazepam 0.5 mg because of her reduced interactions with her family members and apparent low mood. Subsequently, over the next eight weeks, the patient progressively developed manic symptoms for which she presented to our centre.

There had been no previous history of manic or depressive symptoms; however, she had been on diabetes and hypertension medications for 15 years. Her physical examination indicated no abnormalities, while her mental status examination revealed pressured speech, increased psychomotor activity, exalted affect, inflated self-esteem, and impaired judgment. Her haematological investigations were unremarkable except for low haemoglobin (10 mg%). She was diagnosed with late-onset mania and was treated as an outpatient with aripiprazole 10 mg and clonazepam 0.5 mg. Her behavioural symptoms subsided markedly over the next three weeks.

## Discussion

In the elderly, secondary mania is sometimes mistaken as delirium. Mania following a stroke can occur anywhere from immediately after the event to two years later. In comparison to patients with primary mania, those with organic mania may experience cognitive impairment. Post-stroke mania has a clinical profile that is strikingly similar to that of primary mania, including elevated mood/euphoria, rushed speech, ideation, grandiosity, and insomnia. This etiological association is strengthened by the relationship between the stroke and the affective symptoms, not in the presence of other suspected risk factors of mania [6].

Questions like "whether secondary" should be capitalised remain open. However, the low prevalence of late-onset mania has led some to question the usefulness of the term "manic disorders" in the elderly population. In a study, only three out of seven hundred patients experienced mania following a stroke. The majority of the earliest documented cases of secondary mania were linked to lesions in the brain's non-dominant hemisphere. Location defines symptoms better than a neurotransmitter. Left anterior lesions cause significant depression. In late-onset mania, orbitofrontal, thalamic, frontotemporal, and temporal abnormalities are common. The location of the lesion seems to be crucial in secondary manic episodes and decreased inhibition [7].

The risk factors for cardiovascular disease in a sample of 30 inpatients with late-onset bipolar disorder were examined in a retrospective research, with the focus again being on depression rather than mania. Therefore, there is insufficient evidence to conclude whether late-onset mania has an organic or non-organic basis. There hasn't been a lot of research done in this area [8]. Mood stabilisers, conventional antipsychotics like haloperidol, second-generation antipsychotics like olanzapine or risperidone, and benzodiazepines have all been reported to be effective in treating mania following a stroke.

However, this patient was a known case of depressive disorder following MRI findings suggestive of chronic right-sided lacunar infarcts. She was then started on anti-depressants by a private psychiatrist. During the course of her treatment with an anti-depressant i.e., escitalopram, the patient started developing mania symptoms like increased goal-directed activity, flight of ideas, increased psychomotor activity, impaired judgement, irritability, and aggressiveness. The patient was then referred to our hospital in view of the above symptoms. Neither the patient nor any members of the patient's family had a history of mental illness. The patient was prescribed aripiprazole 10 mg and clonazepam 0.5 mg and anti-depressants were immediately stopped following which her symptoms gradually improved.

Late-onset bipolar disorder can be diagnosed only after a thorough investigation into all of the possible contributing factors. Several organic factors, such as co-administration of medicines, infections, metabolic abnormalities, neoplasia, toxins, and vascular infarcts, could make this difficult. Early recognition of post-stroke mania in patients such as ours helps direct appropriate management and minimise risk and suffering. The association between depression and stroke is exceedingly convoluted, and the pathophysiological mechanisms have not been extensively investigated. Physical and psychosocial factors may be significant in the development of post-stroke depression. 

## Conclusions

This case highlights the fact that although rare, mania can present after a chronic brain injury. Therefore, the clinician should be cautious enough in prescribing anti-depressants after evidence of brain injury due to the increased risk of an antidepressant-induced manic switch. Also, any patient with mania who exhibits simultaneous focal abnormalities of the brain and is older than predicted for the development of primary mania should be evaluated for post-stroke mania. Therefore such an atypical presentation can be challenging. It is possible that the triggers for a second manic episode have nothing to do with the ones that caused the first. However, when examining patients with initial bipolar illness using neuroimaging and other methods, the identification of brain lesions that generate secondary mania provides neuroanatomic or neurophysiologic substrates to consider. The several ways in which organic brain pathology might manifest can aid in making a diagnosis, determining the best course of treatment, and evaluating the patient's prognosis. As most chronic organic brain illnesses cannot be cured, leading to poor results and prognosis, a precise diagnosis aided by radiographic research is required.
